# Inhibition, Updating Working Memory, and Shifting Predict Reading Disability Symptoms in a Hybrid Model: Project KIDS

**DOI:** 10.3389/fpsyg.2018.00238

**Published:** 2018-03-20

**Authors:** Mia C. Daucourt, Christopher Schatschneider, Carol M. Connor, Stephanie Al Otaiba, Sara A. Hart

**Affiliations:** ^1^Department of Psychology, Florida State University, Tallahassee, FL, United States; ^2^Florida Center for Reading Research, Florida State University, Tallahassee, FL, United States; ^3^School of Education, University of California, Irvine, Irvine, CA, United States; ^4^Department of Teaching and Learning, Southern Methodist University, Dallas, TX, United States

**Keywords:** reading, reading disability, hybrid model, executive function, shifting, updating, working memory, inhibition

## Abstract

Recent achievement research suggests that executive function (EF), a set of regulatory processes that control both thought and action necessary for goal-directed behavior, is related to typical and atypical reading performance. This project examines the relation of EF, as measured by its components, Inhibition, Updating Working Memory, and Shifting, with a hybrid model of reading disability (RD). Our sample included 420 children who participated in a broader intervention project when they were in KG-third grade (age *M* = 6.63 years, *SD* = 1.04 years, range = 4.79–10.40 years). At the time their EF was assessed, using a parent-report Behavior Rating Inventory of Executive Function (BRIEF), they had a mean age of 13.21 years (*SD* = 1.54 years; range = 10.47–16.63 years). The hybrid model of RD was operationalized as a composite consisting of four symptoms, and set so that any child could have any one, any two, any three, any four, or none of the symptoms included in the hybrid model. The four symptoms include low word reading achievement, unexpected low word reading achievement, poorer reading comprehension compared to listening comprehension, and dual-discrepancy response-to-intervention, requiring both low achievement and low growth in word reading. The results of our multilevel ordinal logistic regression analyses showed a significant relation between all three components of EF (Inhibition, Updating Working Memory, and Shifting) and the hybrid model of RD, and that the strength of EF’s predictive power for RD classification was the highest when RD was modeled as having at least one or more symptoms. Importantly, the chances of being classified as having RD increased as EF performance worsened and decreased as EF performance improved. The question of whether any one EF component would emerge as a superior predictor was also examined and results showed that Inhibition, Updating Working Memory, and Shifting were equally valuable as predictors of the hybrid model of RD. In total, all EF components were significant and equally effective predictors of RD when RD was operationalized using the hybrid model.

## Introduction

Moving away from a focus on general intelligence, achievement research has shifted to an emphasis on other cognitive and behavioral correlates of academic achievement, including self-regulation. One of the main components of self-regulation is a concept originally introduced by Baddeley and Hitch (1974) as the “central executive,” which is currently referred to as “executive function.” Executive function (EF) comprises the skills required for an individual to work toward a goal and make judgments in novel, unforeseen situations and includes regulation of both thought and action. Examples of these self-directed skills include planning ahead, problem solving, decision making, attention maintenance and direction, emotional regulation, and behavioral control (Sesma et al., 2009).

Due to the broad scope of the processes and capacities mediated by EF, there is a lack of consensus among researchers about the specific constituents that make up the EF construct (Sadeh et al., 2012). A significant inquiry about EF is whether EF is a part of a unified construct, like *g* for intelligence, or if it represents a multicomponent system. The unity and diversity paradigm (Miyake et al., 2000; Becker et al., 2014) reconciles this debate by claiming that the EF is both unitary and divisible into subcomponents, which are both inter-related and separate. The shared variance among EF components points to a common thread present in all EF abilities, while the unique variance linked to each individual constituent represents what is distinctive about that particular component of EF (Miyake et al., 2000; Miyake and Friedman, 2012). Research has shown support for EF as an independent yet unitary construct in younger children in both pre-kindergarten and kindergarten (Miyake and Friedman, 2012; Fuhs et al., 2014). On the other hand, research conducted with older children (Brocki and Bohlin, 2004), twins (Friedman et al., 2008), children and adolescents with brain damage (Levin et al., 1996), neurocognitive pathologies (e.g., Culbertson and Zillmer, 1998; Poljac et al., 2010), and typically developing elderly populations (Robbins et al., 1998) has provided evidence for a multicomponent EF system (Lehto et al., 2003; Huizinga et al., 2006). Additionally, many EF tasks that tap presumably separate EFs are not significantly correlated (Miyake et al., 2000; Banich, 2009), which may further indicate the existence of multiple EF constituents.

Even though the precise rudimentary components of EF are still debated, the most common division of EF includes three components: prepotent response inhibition, updating and monitoring of working memory, and mental set shifting (Miyake et al., 2000; Davidson et al., 2006; Best and Miller, 2010; Miyake and Friedman, 2012). “Inhibition” is the capacity to obstruct automatic or dominant responses when they are not appropriate for the context at hand (Miyake et al., 2000; St Clair-Thompson and Gathercole, 2006; Toplak et al., 2013). It includes the ability to suppress the influence of interfering information (Barkley, 1999; Bexkens et al., 2015), and in the case of reading this means being able to suppress the irrelevant meanings of a current word based on the context in which it is nested, or to stop reading at the end of your assigned paragraph when reading aloud in class. “Updating Working Memory” is a screening and coding system that reviews information based on its circumstantial significance, constantly eliminating extraneous information and replacing it with more relevant information. It also represents our cognitive capacity for simultaneous processing of multiple tasks, and in the case of reading, these tasks could include decoding unknown words (Sesma et al., 2009), retrieving the meaning of known words (Sesma et al., 2009), remembering previously read text, and anticipating upcoming text (Daneman and Carpenter, 1980; Sesma et al., 2009; Nouwens et al., 2016). “Shifting” involves back and forth movement between tasks and higher and lower levels of mental processing. It enables us to adapt dynamically to changing task demands and contexts (Deák and Narasimham, 2003; Poljac et al., 2010), and in the case of reading, for example, this could mean mental movement between different verb tenses, known and unknown words, or even between reading environments, such as quietly reading at school versus reading aloud for entertainment at home.

These three components of EF play an important role in learning and memory (McCauley et al., 2010), and have been consistently linked to educational achievement outcomes in reading and math for a variety of age groups (St Clair-Thompson and Gathercole, 2006; McClelland et al., 2007; Foy and Mann, 2013; Becker et al., 2014; Fuhs et al., 2014). For the present study, we will use the theoretically-postulated three component model of EF that includes Inhibition, Updating Working Memory, and Shifting in order to determine the association of EF to reading disability (RD), as well as examine the unique associations of distinctive aspects of EF with RD.

### EF and Reading Disability

Reading difficulties are one of the most pervasive learning impairments found among school-aged children, with 5–10% of students experiencing problems with reading (Compton et al., 2014). Far and beyond all other theories, the prevailing explanation for reading difficulties is a deficit in phonological processing, but recent work has shown that insufficiencies in the EF system may also be underlying deficient reading development (Gombert, 2003; Altemeier et al., 2008; Booth et al., 2010). For example, when accounting for deficiencies in the phonological system, children with RD still have shown diminished performance on tasks assessing EF, such as inhibition and working memory (Swanson et al., 2006; Altemeier et al., 2008; Booth et al., 2010). Indeed, evidence shows that EF deficits are a fundamental feature of RD (Gioia et al., 2002).

The important link between reading and EF lies in the transition from learning to read to reading to learn. At first, the linguistic knowledge necessary for reading is acquired implicitly through regular exposure to patterns in orthography, phonology, and morphology (Gombert, 1992, 2003). This unconscious exposure leads to the creation of a subconsciously-organized and instance-bound linguistic lexicon that includes rudimentary awareness of grapheme to phoneme correspondence (GPC; Gombert, 2003). Then, formal reading instruction begins, ramping up print exposure, while students are taught the explicit rules of GPC. As reading instruction advances, students must be able to take conscious control and monitor their linguistic lexicons in order to respond to unexpected external demands, like reading an unknown word (Gombert, 2003) or properly resolving a conflict between phonology and orthography based on the current context (Bitan et al., 2009). This movement from subconscious to conscious and implicit to explicit is accompanied by a developmental increase in executive control (Gombert, 2003; Bitan et al., 2009). The executive control conferred by EF modulates both top-down and bottom-up processing according to reading task demands (Bitan et al., 2009), which enables readers to discriminate between task-relevant and task-irrelevant information quickly (Bitan et al., 2009) so that reading may become automatic (Gombert, 2003). Without the level of mastery and quick adaptability that EF makes possible, reading difficulties may emerge.

There are many inconsistencies found in the literature exploring the exact role of EF in relation to reading difficulties. According to meta-analytic work, these incongruities can be boiled down to two main moderators: RD definitions and EF task modalities (Stuebing et al., 2002; Booth et al., 2010). Despite the poor 1-year stability of IQ-achievement discrepancy definitions (Schatschneider et al., 2016), they have continued to be one of the most common RD definitions utilized in practice (Spencer et al., 2014), including their use in studies linking EF and RD (e.g., Altemeier et al., 2008). Based on the results from two meta-analyses (Stuebing et al., 2002; Booth et al., 2010), the association of EF to IQ-achievement discrepancy definitions of RD shows lower mean effect sizes than the association of EF to non-discrepancy definitions of RD (Booth et al., 2010). One possible explanation for this difference is that IQ-discrepant readers are no different than IQ-consistent readers, and that the RD definition used only appears to matter because of differences in EF and IQ task modality (Booth et al., 2010). For example, verbal versus non-verbal IQ may yield different results when included with EF in an IQ-discrepant RD framework, especially depending on whether a verbal or non-verbal EF task is used. Since a majority of EF tasks incorporate a verbal component (e.g., Altemeier et al., 2008), it is difficult to parse out whether the driving force behind the task performance is truly EF or the phonological or verbal processing needed to complete the task.

In response to the confusion presented in the literature examining the role of EF in reading achievement due to EF task modality and RD definitions, we implemented two main techniques in the present study to assist in drawing clearer conclusions about the association between EF and RD. First, we measured EF with the Behavior Rating Inventory of Executive Function (BRIEF), which is a non-verbal instrument that captures EF as it is manifested behaviorally. By employing a non-verbal EF measure, we are able to avoid the uncertainty about the contributing role of verbal processing to EF performance. In addition, most of the work on EF and RD thus far has used cognitive indices of EF ability (e.g., Altemeier et al., 2008), which have been shown to correlate poorly with behavioral EF measures (e.g., McCauley et al., 2010), so our use of the BRIEF subscales in predicting RD may yield interesting new results. Second, in order to avoid the potential pitfalls embedded in the use of IQ-discrepant RD definitions, we employed a hybrid model approach to RD classification, the benefits of which we discuss in more detail in the following section.

### Hybrid Models for RD Classification

A promising solution to the low reliability of IQ discrepancy-based and other single-criterion RD models, and their inconsistent association with EF, is the implementation of hybrid models for RD classification (Wagner, 2008; Waesche et al., 2011; Fletcher et al., 2013; Spencer et al., 2014). Historically, RD models have relied on a single benchmark, which is most commonly based on either IQ-achievement discrepancy (Bateman, 1965), cognitive discrepancy (Stuebing et al., 2012), or response-to-intervention (RTI) or instruction (Fuchs and Fuchs, 2006) definitions. Hybrid models take a multi-component approach to RD measurement, which makes them comparatively more stable than other RD classification techniques (Spencer et al., 2014; Schatschneider et al., 2016). Fundamentally, the hybrid model approach to RD classification is based on the idea that a construct is more precisely captured by measuring it in many ways. As such, the hybrid model employed in the present study defined RD as a latent construct made up of four measured symptoms that are described in more detail below.

A recent publication by Spencer et al. (2014) examined the 1- and 2-year stability of a hybrid model approach to RD classification utilizing four indicators of RD that were all chosen based on traditional RD definitions. The RD indicators included low achievement in word reading, unexpected low achievement in word reading, poorer reading comprehension compared to listening comprehension, and a dual-discrepancy RTI model that necessitated both low achievement and low growth in word reading. In this version of the hybrid model, RD was characterized using a symptom approach, similar to the method employed in the Diagnostic and Statistical Manual of Mental Disorders (5th ed.; DSM-V; American Psychiatric Association, 2013). The symptoms were calculated utilizing cutoff points ranging in severity from the 3rd to the 25th percentile, and regardless of which symptom was examined, results revealed that severity and stability were inversely related, with the highest cutoff points (i.e., the 25th percentile), yielding the most stable results for RD classification. In the same vein, Schatschneider et al. (2016) conducted a simulation study that found that hybrid models that incorporated many symptoms of RD, instead of any one RD benchmark, provided the most stable classification scheme for RD, and that the 25th percentile was also the most stable cutoff point for each symptom in a constellation (i.e., multi-symptom) model. The findings of these two investigations, as well as the results yielded by a similar study conducted by Waesche et al. (2011), provide clear evidence for the advantages of using a hybrid model that classifies RD as a latent construct made up of many measured symptoms of RD as the most reliable and state of the science approach to RD classification. Additionally, the methods and findings outlined by these papers (Waesche et al., 2011; Spencer et al., 2014; Schatschneider et al., 2016) clearly point to the 25th percentile as the best cutoff point for achieving the highest reliability in RD symptom identification within a hybrid model. Accordingly, a 25th percentile cut was utilized for the calculation of each RD symptom in the hybrid model utilized in the present study. Next, we will take a closer look at the each of the four hybrid model symptoms. The first symptom, low achievement in word reading, represents the simplest conceptualization of RD, and is based on the fact that students who fail to reach a certain level of word reading performance at the end of the school year are likely to be reading disabled and require some form of additional intervention. Unexpected low achievement, an IQ-achievement discrepancy definition for RD, was operationalized as unexpectedly low word reading achievement based on verbal aptitude. This symptom was modeled after the idea that a student who demonstrates a certain capacity in his/her general intelligence should be able to translate that same capacity into all domains, including reading achievement. Our reason for focusing on word reading in these initial two symptoms was that phonological awareness, which underlies word reading ability, serves as a precursor to more advanced reading skills (e.g., Holloway et al., 2015), so it provides a useful early indicator of reading difficulties before reading demands become more advanced (Fletcher et al., 2007; Spencer et al., 2014).

In an effort to differentiate between reading-specific deficits and general insufficiencies in overall cognitive processing the hybrid model also included a symptom based on a cognitive discrepancy definition of RD (Torgesen, 2002; Spencer et al., 2014). Cognitive discrepancy refers to a situation in which a student’s achievement in one cognitive domain outperforms his/her achievement in another cognitive domain. More specifically, the symptom was defined as poorer reading comprehension compared to listening comprehension performance. The advantage of this symptom is that it picks up on students that may not be performing poorly in general, but are failing to achieve at the same level in reading as they are in other domains.

Finally, the dual-discrepancy RTI RD symptom requires two elements for qualification: low word reading growth over the school year in conjunction with low end-of-the-year word reading performance (Schatschneider et al., 2016). This symptom is based on the fact that when a student receives direct reading instruction in a classroom and fails to grow or reach a certain level of reading achievement it is an indication that the child has failed to respond to intervention or instruction (Fuchs et al., 2002; Spencer et al., 2014; Schatschneider et al., 2016).

### Specific EF Components and Reading Disability

Past research reveals mixed findings on the differential role of specific EF components associated with RD (e.g., Swanson, 2003; Swanson et al., 2006; Booth et al., 2010; Sáez et al., 2012). There is a general consensus that inhibition is a fundamental element of executive processing, which both allows for the development, and also constrains the performance, of all other executive functioning components (Miyake et al., 2000; Foy and Mann, 2013). On its own, inhibitory ability may be especially important for early reading skills, like processing or making judgments about phonemes (Foy and Mann, 2013). In the case of working memory, poor inhibitory skills are likely to lead to intrusion errors (DeBeni et al., 1998; Foy and Mann, 2013) and the expression of inappropriate responses or guesses (Stevens et al., 2009; Foy and Mann, 2013), and for shifting, poor inhibition will likely result in representational inflexibility, such as an over-reliance on sight word reading (Diamond, 2002). All of these scenarios may create circumstances in which reading difficulties and errors are more likely (Reiter et al., 2005; Altemeier et al., 2008). For example, performance on the Stroop task, a test of inhibitory ability, is diminished in children with reading difficulties (Everatt et al., 1997; Booth et al., 2010). However, as a task that requires reading, the Stroop task may be revealing reading difficulties unrelated to EF. Even though some work has failed to find a significant difference between typically-developing and RD readers on tests of inhibitory control (Bexkens et al., 2015), Reiter et al. (2005) found that children with RD were impaired on inhibitory tasks in their processing time and error correction abilities and were more likely to commit more errors overall. These results provide support for similar findings of specific inhibitory decrements in children with reading difficulties (DeBeni et al., 1998; Altemeier et al., 2008), even when controlling for age, short-term memory, and vocabulary (Foy and Mann, 2013). Further research into the specific relation between inhibition and RD would contribute to resolving these inconsistent findings.

Working memory is the most extensively explored of the EFs (e.g., Swanson, 2003; Pickering and Gathercole, 2004; Reiter et al., 2005; Gathercole et al., 2006; Cutting et al., 2009; Kieffer et al., 2013), but its relation with RD is still not fully understood. While the original conceptualization of EF by Baddeley and Hitch (1974) was divided into a three-part system comprised of the phonological loop, the visuospatial sketchpad, and the central executive, the results yielded by modern factor analytic work (e.g., Miyake et al., 2000) have transformed the contemporary operationalization of EF by creating a working memory component (along with inhibition and shifting components; Miyake et al., 2000). Most commonly, the phonological loop and visuospatial sketchpad are either collapsed into a singular working memory construct (e.g., Cutting et al., 2009), or re-conceptualized as verbal working memory and non-verbal working memory, respectively (e.g., Gathercole et al., 2006). Importantly, studies have found a vital link between working memory and literacy skills, whether working memory was operationalized singularly (Cutting et al., 2009) or divided into its verbal and non-verbal parts (Gathercole et al., 2006). There is evidence that verbal working memory may be especially important for reading-related skills (St Clair-Thompson and Gathercole, 2006; Foy and Mann, 2013), along with evidence that working memory, as a singular measure, also supports reading comprehension and reading fluency growth in school-aged children (Swanson and Jerman, 2007). Given the evidence for a significant relation between reading outcomes and working memory, regardless of which conceptualization was employed, we chose to utilize a single-component definition for working memory in the present study.

Looking specifically at reading difficulties, the relations found with working memory and RD are also mixed. In a study of 6- to 49-year-olds that examined the relation of working memory and reading difficulties, working memory deficits were present in individuals with reading difficulties across all ages (Chiappe et al., 2000). Even when accounting for potentially confounding variables by including a battery of tasks for related cognitive skills, RD readers have still demonstrated working memory impairments (Reiter et al., 2005). In some cases, the association of working memory and (word-level) reading difficulties has lacked a significant relation because EF deficits can be fully accounted for by shortcomings in decoding (Sesma et al., 2009) or phonological processing (Locascio et al., 2011), while other studies have shown that poor reading performance cannot be fully attributed to insufficiencies in the phonological system (Swanson, 2003; Swanson et al., 2006). In fact, reading success is most likely the product of both phonological processing skills and the supportive role played by updating working memory (Iglesias-Sarmiento et al., 2015). Beyond phonological processing, studies that account for additional cognitive abilities, like intelligence, still find suppressed working memory task performance in children with reading difficulties (e.g., Gathercole et al., 2006; Swanson et al., 2006). These findings may be explained by the fact that working memory capacity constrains an individual’s ultimate level of proficiency in any academic realm, including reading, by serving as a limiting factor on the amount of knowledge and skill an individual can ultimately acquire (Gathercole et al., 2006). It stands to reason that individuals who demonstrate difficulties in reading may simply have a low working memory capacity that limits their capability for reading skill acquisition. By including Updating Working Memory in our model of EF we hope to further elucidate its role in reading disabilities.

Shifting, the third component of the EF model proposed by Miyake et al. (2000), is the most under-explored of the EFs, and findings about its role in reading performance are still conflicting (Stoet et al., 2007). There is some evidence that shifting may be a weaker predictor of reading skills deficits (Bierman et al., 2008) and early literacy skills (e.g., Foy and Mann, 2013) than inhibition and working memory. In fact, some investigators posit that shifting is simply an expansion of inhibitory control and its interaction with attention, and not a separable skill (Diamond, 2002; Diamond et al., 2005). Although others have found a specific role for shifting in processing linguistic information (Wolf et al., 1986), recent neurological work utilizing EEG technology has found that children with RD do not show impaired performance on shifting tasks when compared with typically developing controls (Horowitz-Kraus, 2014). On the contrary, Poljac et al. (2010) found a shifting-specific delay in RD children but not in autistic children. When considering these conflicting results, there is an obvious need for further exploration of the role played by shifting for children with reading difficulties.

### Present Study

Taken together, these findings suggest that there is a relation between executive functioning and RD. Overall, EF and its component skills contribute to reading by helping students organize, recall, and integrate new and existing information, but the details of the specific relation between EF and RD are still mixed. Furthermore, work examining the association of EF with RD has not previously used a hybrid model approach for defining RD, which is a more comprehensive and modern definition of RD than single-criterion models. In this paper, we will examine the relation of the three-component model of EF, which includes Inhibition, Updating Working Memory, and Shifting (Miyake et al., 2000; Booth et al., 2010; Nouwens et al., 2016), with the hybrid model of RD (Spencer et al., 2014; Schatschneider et al., 2016). Moreover, we will explore the predictive strength of each EF component skill in order to determine whether one EF is more important for RD identification or not. Our first research question was “How does EF predict RD classification in a hybrid model of RD?” Our second research question was “Is one EF component more important than the others for RD classification?”

## Materials and Methods

### Participants

The participants in this study were 420 children (51.20% female) who participated in Project KIDS. Project KIDS had two components. The first component involved combining data, using integrative data analysis (IDA), from eight completed literacy and math randomized-control trial intervention projects that occurred in north Florida schools at some point during the 2005–2006 to the 2012–2013 school years (Connor et al., 2007, 2011a,b, 2013; Al Otaiba et al., 2011a,b, 2014a,b). This data integration resulted in a dataset of literacy, math, and related achievement tests of 3868 children, which then served as the population to draw from for the second component of Project KIDS. This second component involved an extensive parental questionnaire, including a parental report of EF. During the spring and summer of 2014, questionnaires were mailed to the last known addresses of the original intervention participants’ families. The final sample size for the second component of Project KIDS was *n* = 445, however only *n* = 420 had EF data available, so those 420 participants were moved forward into all analyses.

Given the low response rate for the questionnaire portion of Project KIDS, comparisons of the differences between the original population (*n* = 3868) and the sample of this current report (*n* = 420) were done for the achievement measures used in this report, as well as on demographic information. There were significant differences between the groups for word reading [*t*(3315) = 3.46, *p* < 0.01; original population *M* = 35.52, *SD* = 11.88, *n* = 2946; report sample *M* = 37.77, *SD* = 11.21, *n* = 371], reading comprehension [*t*(3320) = 3.45, *p* < 0.01; original population *M* = 17.94, *SD* = 7.47, *n* = 2942; report sample *M* = 19.34, *SD* = 7.17, *n* = 380], and vocabulary [*t*(3348) = 4.11, *p* < 0.0001; original population *M* = 20.10, *SD* = 3.49, *n* = 2977; report sample *M* = 20.89, *SD* = 3.53, *n* = 373]. There were no significant differences noted between the original population and the current report sample for age [*t*(3864) = -1.36, *p* = 0.18], sex [χ^2^(1) = 1.42, *p* = 0.23], and race-ethnicity [χ^2^(1) = 0.89, *p* = 0.35], although there was a significant difference for free and reduced lunch status, with the questionnaire sample showing fewer students qualified for free or reduced lunch [χ^2^(1) = 4.67, *p* = 0.03].

The original intervention projects occurred when the children were in kindergarten, first, second, or third grade (age *M* = 6.63 years, *SD* = 1.04 years, range = 4.79–10.40 years), although at the time of questionnaire completion, the participants had a mean age of 13.21 years (*SD* = 1.54 years; range = 10.47–16.63 years). The demographic distribution of the current sample included 56.56% White, 35.08% Black/African American, 5.73% Other or Mixed children. Parental informed consent in writing was obtained for all participants in Project KIDS. The Florida State University Institutional Review Board approved all aspects of Project KIDS.

### Measures

One caregiver of the original intervention project children (88% biological mother responded) was asked to complete a questionnaire either by mail or online, using Qualtrics. This questionnaire asked about the parents’ basic demographic information, such as age, education level, occupation, household income, ethnicity, and race and about the siblings of the child involved in the study, including their age, gender, and relationship to the participant. The questionnaire also included a section on family medical history that asked about learning difficulties and learning disability diagnoses, and a series of questionnaires concerning the home environment, child’s behaviors (including the BRIEF), nutrition, and sleep habits. All children completed a large battery of cognitive ability and achievement measures during the original intervention projects’ protocols, usually administered three times during the original intervention year, early fall, winter (early spring semester), and late spring semester.

#### Behavior Rating Inventory of Executive Function

The parent form of the BRIEF (Gioia et al., 2002) is an 86-item questionnaire that assesses the EFs of children. Parents were asked to read a list of statements that describe their child and report on whether their child had problems with the listed behaviors over the past 6 months using a 3-point scale (Never, Sometimes, Often). Each item loads onto one of eight scales (Inhibit, Shift, Emotional Control, Initiate, Working Memory, Plan/Organize, Organization of Materials, and Monitor), which combine into two summary measures and one composite score. The goal of these indices is to detect possible deficiency in one or more areas of EF based on child behavior (high BRIEF scores correspond to low executive functioning; McCauley et al., 2010). For the present report, the Working Memory, Shift, and Inhibit scales were used. Reliabilities in this sample for all three were good (Cronbach’s alphas: Inhibition = 0.93, Updating Working Memory = 0.92, Shifting = 0.87).

#### Woodcock–Johnson III Tests of Achievement Letter–Word Identification

The Woodcock–Johnson III (WJ) Tests of Achievement Letter–Word Identification subtest (LWID; Woodcock et al., 2007) is a norm-referenced standardized measure. It is comprised of 75 items that measure reading decoding, or the ability to visually recognize word forms or use phonological ability to pronounce words associated with word forms. Published median split-half reliability for the LWID is 0.94 (Schrank et al., 2001).

#### Woodcock–Johnson III Tests of Achievement Picture Vocabulary

The WJ Picture Vocabulary subtest (PV; Woodcock et al., 2007), which measures expressive language through picture naming, was used to assess children’s vocabulary. The test–retest reliability on this test falls in a range of 0.70–0.81 (Schrank et al., 2001), and the assessment includes 44 items.

#### Woodcock–Johnson III Tests of Achievement Passage Comprehension

The WJ Passage Comprehension subtest (PC; Woodcock et al., 2007) is used to measure written text comprehension through matching of pictures with words and phrases and fill-in-the-blank sentences and paragraphs of increasing complexity. For ages 5–19, its median reliability is 0.88 (Schrank et al., 2001), and the assessment includes 47 items.

### Data Analytic Plan

Prior to analyses specific to this paper, IDA (Curran et al., 2008, 2014) was used to combine all eight intervention projects’ early fall (pre-intervention) and late spring (post-intervention) LWID data. At the heart of IDA lies measurement invariance modeling. Measurement invariance modeling in IDA is a disciplined approach to combining datasets from multiple projects. IDA involves using a moderated non-linear factor analysis (MNLFA), which allows for raw item-level data to be combined across projects, modeling potential sources of heterogeneity (e.g., sampling, age/grade) using differential item functioning (DIF). In this case, the MNLFA was the equivalent of a 2-PL model with project, and age (both linear and quadratic terms) DIF modeled. As recommended by Curran et al. (2014), we randomly selected one time point per student for a calibration sample, and also pruned any item that did not have at least 5% coverage of responses (resulting in LWID items 11–75 being included). Using the calibration sample, we first tested for DIF on the factor mean and variance. Second, we tested for DIF on each item intercept and loadings, accounting for factor DIF. Any non-significant DIF for a parameter was constrained to equality. After the final model was settled, the full data was run using code where the final beta weights were fixed, and the factor score was saved out as the new LWID score for both time points for each child. All analyses were conducted in Mplus 7.3 (Muthén and Muthén, 1998–2012). After conducting IDA on LWID, we found that the new IDA LWID factor scores and the previous simply combined LWID raw total scores were correlated at *r* = 0.97 (early fall) and *r* = 0.99 (late spring). We believe these high correlations are the result of the WJ tests being developed using Item Response Theory models for their scoring and having standardized administration. The original project staff for all projects were very experienced, and the children were relatively close in age and geographic region, meaning that chances for DIF were minimized. Given the computational time for doing the IDA was very large (weeks of run time on a dedicated server) and the correlations between raw score and ability score were so high, we decided to use the raw total scores for all the WJ measures.

Since the hybrid model was based on the WJ LWID, PV, and PC assessments administered in the original project, and some assessment data were missing, we first conducted multiple imputation (Rubin, 1987) to avoid case-wise deletion and enable all analyses to be conducted on a full data set with no missing values. Multiple imputation requires that all variables be normally distributed, and inspection of descriptive statistics confirmed that this assumption was met (see **Table 1**; skewness and kurtosis between ±2; Tannenbaum et al., 2009). Prior to performing the imputation, students missing all data for the assessments needed for symptom calculation were dropped from the sample (*n* = 168, 4% of overall sample), since they had no achievement data on which to estimate replacement values, resulting in a drop in the sample size from 4036 to 3868. As a next step, we assessed the missingness of each of our variables of interest and found that no variable was missing more than 14.19% of data (see **Table 1**). Additionally, a Shifting score was missing for one of the Project KIDS questionnaire participants, so Shifting was also included in the imputation model in order to replace the missing value. Finally, Proc MI (multiple imputation) in SAS 9.4 was used to impute 20 datasets based on the covariance matrix of all available data. The resulting 20 data sets were combined, and a mean score of all 20 data points was calculated for each missing value to replace previously missing data points. Pre-imputation descriptive statistics are presented in **Table 1**, and post-imputation descriptives are presented in **Table 2**. The tables show that the means and standard deviations before and after imputation are comparable, and that the data moving forward after multiple imputation are complete, with no missing values for the EF subscales or the WJ assessments used to calculate the hybrid model symptoms.

**Table 1:** Pre-imputation descriptive statistics.

	*N*	*N*_miss_	Mean	Median	Min	Max	*SD*	Skew	Kurtosis
**WJ assessments**									
Fall LWID	3868	0	25.35	23.00	0.00	68.00	13.06	0.47	-0.56
Winter LWID	3470	398	32.25	32.00	2.00	69.00	12.24	0.09	-0.73
Spring LWID	3319	549	35.77	37.00	4.00	71.00	11.83	-0.15	-0.67
Fall PV	3847	21	18.57	18.00	0.00	34.00	3.62	-0.17	0.98
Winter PV	3480	388	19.53	20.00	5.00	34.00	3.62	-0.15	0.49
Spring PV	3352	516	20.19	20.00	4.00	35.00	3.50	0.06	0.39
Fall PC	2999	869	14.29	14.00	0.00	36.00	7.66	0.25	-1.01
Winter PC	2041	1827	16.31	18.00	0.00	38.00	7.68	-0.19	-0.97
Spring PC	3324	544	18.10	19.00	0.00	35.00	7.45	-0.26	-0.80
**Executive functioning**									
Inhibition	420	3448	13.95	12.00	10.00	30.00	4.42	1.24	1.03
Updating WM	420	3448	16.05	15.00	10.00	30.00	4.84	0.64	-0.29
Shifting	419	3449	12.13	11.00	8.00	24.00	3.54	0.80	0.09


*All values reflect raw scores prior to standardizing. *N*_*miss*_, number of missing observations; Updating WM, Updating Working Memory; WJ assessments, Woodcock–Johnson III Tests of Achievement; LWID, Letter–Word Identification subtest; PV, Picture Vocabulary subtest; PC, Passage Comprehension subtest.*

**Table 2:** Post-imputation descriptive statistics.

	*N*	*N*_miss_	Mean	Median	Min	Max	*SD*	Skew	Kurtosis
**WJ assessments**									
Fall LWID	3868	0	25.35	26.00	0.00	68.00	13.06	0.47	-0.56
Winter LWID	3868	0	31.93	35.00	2.00	69.00	12.22	0.13	-0.74
Spring LWID	3868	0	35.37	38.00	4.00	71.00	11.78	-0.08	-0.71
Fall PV	3868	0	18.56	19.00	0.00	34.00	3.62	-0.17	0.98
Winter PV	3868	0	19.42	20.00	5.00	34.00	3.61	-0.15	0.54
Spring PV	3868	0	20.02	21.00	4.00	35.00	3.50	0.02	0.53
Fall PC	3868	0	12.99	14.00	-2.98	36.00	7.63	0.41	-0.85
Winter PC	3868	0	16.01	18.28	-1.65	38.00	7.52	-0.05	-0.91
Spring PC	3868	0	17.82	20.00	-1.32	35.69	7.42	-0.18	-0.83
**Executive functioning**									
Inhibition	420	3448	13.95	12.00	10.00	30.00	4.42	1.24	1.03
Updating WM	420	3448	16.05	15.00	10.00	30.00	4.84	0.64	-0.29
Shifting	420	3448	12.13	11.00	8.00	24.00	3.54	0.80	0.10


*All values reflect raw scores prior to standardizing. *N*_*miss*_, number of missing observations; Updating WM, Updating Working Memory; WJ assessments, Woodcock–Johnson III Tests of Achievement; LWID, Letter–Word Identification subtest; PV, Picture Vocabulary subtest; PC, Passage Comprehension subtest.*

The hybrid model of RD was operationalized following Spencer et al. (2014) and Schatschneider et al. (2016), where students were categorized as having any one, two, three, or four symptoms of RD (modeling the “ANYn” categorization in Spencer et al., 2014). The four symptoms of RD included low word reading achievement, unexpected low word reading achievement, poorer reading comprehension compared to listening comprehension, and a dual-discrepancy RTI model that required both low growth and low achievement in word reading. All symptoms were calculated using the full sample of achievement data (*n* = 3868) in SAS 9.4. Low achievement was operationalized as any score below the 25th percentile on spring word reading scores. Unexpected low achievement, an IQ-achievement discrepancy definition for RD, was operationalized as unexpectedly low word reading achievement based on verbal aptitude. It was calculated by residualizing spring word reading scores on spring vocabulary scores (a proxy for verbal aptitude) and implementing a 25th percentile cut. Poorer reading comprehension compared to listening comprehension captured the cognitive discrepancy definition of RD, and was calculated by residualizing spring PC scores on spring PV scores (a proxy for listening comprehension; Senechal et al., 2006; Spencer et al., 2014). The dual-discrepancy RTI symptom, as its name suggests, required two parts and was calculated with a 25th percentile cut on the slopes (i.e., growth) and intercepts (i.e., end-of-the-year score) of each child’s residualized gains in word reading. After the calculation of these four symptoms, children were assigned a value of 0 (i.e., not showing any symptom), 1 (showing any one symptom), 2 (showing any two symptoms), 3 (showing any three symptoms), or 4 (showing all four symptoms).

#### Research Question 1

Using the hybrid model symptoms assignment described above, we determined if the EF measures predicted RD using three proportional odds models for ordinal logistic regression analyses in a hierarchical linear modeling (HLM) framework. EF data were only available for students that participated in the second part of Project KIDS, the questionnaire follow-up study, so the full sample size in all analyses utilizing EF was 420. Since many children from the same classroom were part of the original interventions (and therefore were in this sample), HLM was used to control for teacher-level variance and account for any teacher effects. The use of proportional odds models was necessary in order to extend the standard binary logistic model to account for a response variable, like the hybrid model of RD, that had ordered categories (i.e., having four symptoms is worse than having three symptoms; Brant, 1990). Although the hybrid model is not set up so that any one symptom is considered more important or severe than any other, having more than one RD symptom qualifies as more severe RD because the child would be demonstrating difficulties in multiple reading domains. In a proportional odds model, the event being modeled, which in this case was RD status in the hybrid model of RD, is the outcome of being classified in a particular category or any later category, and in our analyses, any later category represents one additional symptom of RD. For instance, when Inhibition was used to predict RD status in the case of the three-symptom group, the model predicted the likelihood of being classified as having any three or four symptoms of RD. In predicting the two-symptom group, Inhibition predicted the likelihood of classification into the two-, three-, or four-symptom group, and when predicting the one-symptom group, Inhibition predicted the likelihood of classification into the one-, two-, three-, or four-symptom group. Only when predicting the four-symptom group, was the outcome independent from other groups. Three different proportional odds models were run, one for each EF component, predicting our composite measure of RD that included all four symptoms of the hybrid model. First, Inhibition was used to predict RD status, which could be defined as any one, any two, any three, or any four symptoms of RD from the hybrid model. Subsequently, Updating Working Memory was used to predict RD status, and finally, Shifting was used to predict RD status. This was done using Proc Glimmix in SAS 9.4.

#### Research Question 2

To answer our second inquiry, we conducted a Profile Analysis, controlling for teacher-level variance, to examine whether there were significant differences in the association of each of the EF components with each RD symptom group (one, two, three, or four symptoms of RD). In other words, we were interested in not only determining to what extent EF predicted RD status, but also, when multiple EF components predicted RD status, which EF was the best predictor. Although Profile Analysis has a series of proposed models in the model building process, for this analysis we utilized the “flatness” test. The flatness test is used to establish whether one point on a line has a significantly different mean than any other point on the same line. If the points do not differ significantly, the line is considered statistically flat, indicating that no one point on that line is a better predictor than any other point on that line. In this case, there were four lines, each representing one of the four RD symptom groups (i.e., one-symptom RD group, two-symptom RD group, etc.), and each line had three points, one for each of the three EF components (one for Inhibition, one for Updating Working Memory, and one for Shifting). This analysis was conducted using Proc Mixed in SAS 9.4.

## Results

### Descriptives

Descriptive statistics for all EF components and WJ assessments used in subsequent analyses are displayed in **Table 2**. These values reflect unstandardized values and the BRIEF scores before reverse-scoring, so that high scores on any BRIEF subscale represented weaker executive functioning. **Table 3** shows the Pearson and Spearman correlation coefficients for the three components of EF, the WJ assessments, and the hybrid model symptoms and RD groups. Prior to calculating the correlations, all scores were standardized, and, for ease of interpretation, BRIEF scores were reversed so that high scores reflected high executive functioning. The correlations of the EF components indicated that they are separable indices (*r* = 0.59–0.65, *p* < 0.0001). Inhibition and Shifting were significantly correlated with all four hybrid model symptoms separately, but Updating Working Memory was only significantly associated with two of the four hybrid model symptoms, namely low word reading achievement and dual-discrepancy RTI in word reading. All three EF components were significantly negatively correlated with the hybrid model of RD variable, indicating that higher EF was associated with having fewer symptoms of RD. **Table 4** displays the frequency of students identified in the one-, two-, three, and four-symptom RD groups of the hybrid model as well as the frequency of students exhibiting each specific symptom.

**Table 3:** Correlations for all included variables.

	1	2	3	4	5	6	7	8	9	10	11	12	13
1. Inhibition	–												
	–												
2. Updating Working Memory	0.64												
	<0.0001												
3. Shifting	0.59	0.64											
	<0.0001	<0.0001											
4. Low achievement	-0.17	-0.17	-0.17										
	0.0005	0.0004	0.0006										
5. Unexpected low achievement	-0.12	-0.07	-0.11	0.61									
	0.0125	0.1813	0.0242	<0.0001									
6. RC < LC	-0.11	-0.07	-0.11	0.61	0.68								
	0.0213	0.1286	0.0216	<0.0001	<0.0001								
8. Dual-discrepancy RTI	-0.19	-0.16	-0.15	0.35	0.27	0.19							
	<0.0001	0.0008	0.0015	<0.0001	<0.0001	<0.0001							
9. Hybrid Model of RD	-0.18	-0.14	-0.16	0.85	0.87	0.85	0.45						
	0.0003	0.0048	0.0007	<0.0001	<0.0001	<0.0001	<0.0001						
10. Fall LWID	0.19	0.16	0.13	-0.63	-0.48	-0.48	-0.24	-0.61					
	0.0001	0.0014	0.0059	<0.0001	<0.0001	<0.0001	<0.0001	<0.0001					
11. Fall PV	0.22	0.22	0.20	-0.48	-0.15	-0.20	-0.27	-0.34	0.61				
	<0.0001	<0.0001	<0.0001	<0.0001	0.0024	<0.0001	<0.0001	<0.0001	<0.0001				
12. Fall PC	0.19	0.14	0.13	-0.62	-0.46	-0.45	-0.24	-0.59	0.93	0.63			
	0.0001	0.0038	0.0103	<0.0001	<0.0001	<0.0001	<0.0001	<0.0001	<0.0001	<0.0001			
13. Spring LWID	0.22	0.22	0.21	-0.77	-0.59	-0.56	-0.39	-0.76	0.90	0.63	0.86		
	<0.0001	<0.0001	<0.0001	<0.0001	<0.0001	<0.0001	<0.0001	<0.0001	<0.0001	<0.0001	<0.0001		
14. Spring PV	19	0.20	0.15	-0.50	-0.07	-0.12	-0.25	-0.29	0.64	0.82	0.64	0.66	
	<0.0001	<0.0001	0.0016	<0.0001	0.1723	0.0114	<0.0001	<0.0001	<0.0001	<0.0001	<0.0001	<0.0001	
15. Spring PC	0.21	0.20	0.19	-0.75	-0.50	-0.62	-0.33	-0.72	0.82	0.67	0.82	0.90	0.70
	<0.0001	<0.0001	<0.0001	<0.0001	<0.0001	<0.0001	<0.0001	<0.0001	<0.0001	<0.0001	<0.0001	<0.0001	<0.0001


**N* = 420; for each variable, the first number represents the Pearson correlation coefficient and the second number represents the exact *p-*value; all correlations that involved the Hybrid Model of RD were calculated as Spearman correlation coefficients instead. RC < LC, poorer reading comprehension compared to listening comprehension; LWID, Letter–Word Identification subtest; PV, Picture Vocabulary subtest; PC, Passage Comprehension subtest.*

**Table 4:** Frequency of students identified by the hybrid model of RD.

	Number of students	%
**Number of RD symptoms**		
None	281	66.90
One	49	11.67
Two	29	6.90
Three	52	12.38
Four	9	2.14
**Type of RD symptom**		
Low achievement	93	22.14
Unexpected low achievement	99	23.57
RC < LC	93	22.14
Dual-discrepancy RTI	14	3.33


**N* = 420. RC < LC, poorer reading comprehension compared to listening comprehension.*

### Primary Analyses

#### Research Question 1: How Does EF Predict RD Classification in a Hybrid Model of RD?

The proportional odds models for hierarchical ordinal logistic regression indicated that, while controlling for teacher-level variance, Inhibition (OR = 0.74, 95% CI = 0.58, 0.94), Updating Working Memory (OR = 0.78, 95% CI = 0.62, 0.99), and Shifting (OR = 0.78, 95% CI = 0.60, 0.96) were all significantly related to the hybrid model of RD for students that exhibited all four symptoms of RD, and students who demonstrated any three or four, any two, three, or four, or any one, two, three, or four symptoms of RD (see **Table 5**). According to the proportional probabilities, those students at the mean level of Inhibition ability have a 31% chance of being classified as having one, two, three, or four symptoms of RD, while those students at one standard deviation above the mean of Inhibition (i.e., higher EF than average) have a 26% chance of being classified as having one, two, three, or four symptoms of RD, and those students functioning one standard deviation below the mean of Inhibition (i.e., lower EF than average) have a 40% chance of being classified as having one, two, three, or four symptoms of RD. When predicting the two-symptom RD group, students at the mean level of Inhibition ability have a 16% chance of being classified as having two, three, or four RD symptoms, while students one standard deviation above the mean and one standard deviation below the mean of Inhibition have a 12% and 20% likelihood, respectively, of being classified as having two, three, or four symptoms of RD. When Inhibition is used to predict classification in the three-symptom RD group, students at the mean functioning of Inhibition have a 9% chance of being classified as having three or four symptoms of RD, while students one standard deviation above, and one standard deviation below the mean of Inhibition have a 7% and 11% chance, respectively, of being classified as having three or four symptoms of RD. Finally, when utilizing Inhibition to predict the four-symptom RD group, the likelihood that students will be classified as having all four symptoms of RD is 1% for all levels of Inhibition.

**Table 5:** Hierarchical ordinal logistic regression results.

	-1 *SD* PP (Coeff)	Mean PP (Coeff)	+1 *SD* PP (Coeff)	SE	*t*	*p*-value	OR (95% CI)
**Inhibition**							
1+ RD symptoms	0.38 (-4.79)	0.31	0.26	0.40	-11.86	<0.0001	
2+ RD symptoms	0.20 (-2.36)	0.16	0.12	0.21	-11.44	<0.0001	
3+ RD symptoms	0.11 (-1.68)	0.09	0.07	0.19	-9.03	<0.0001	
4 RD symptoms	0.01 (-0.78)	0.01	0.01	0.17	-4.58	<0.0001	
Constant	–	-0.30	–	0.12	-2.46	0.0147	0.74 (0.58, 0.94)
**Updating WM**							
1+ RD symptoms	0.37 (-4.77)	0.31	0.26	0.40	-11.85	<0.0001	
2+ RD symptoms	0.19 (-2.36)	0.16	0.13	0.21	-11.47	<0.0001	
3+ RD symptoms	0.11 (-1.69)	0.09	0.07	0.19	-9.06	<0.0001	
4 RD symptoms	0.01 (-0.79)	0.01	0.01	0.17	-4.61	<0.0001	
Constant	–	-0.24	–	0.12	-2.03	0.0440	0.78 (0.62, 0.99)
**Shifting**							
1+ RD symptoms	0.38 (-4.78)	0.31	0.26	0.40	-11.85	<0.0001	
2+ RD symptoms	0.20 (-2.37)	0.16	0.12	0.21	-11.50	<0.0001	
3+ RD symptoms	0.11 (-1.69)	0.09	0.07	0.19	-9.09	<0.0001	
4 RD symptoms	0.01 (-0.79)	0.01	0.01	0.17	-4.64	<0.0001	
Constant	–	-0.28	–	0.12	-2.30	0.0220	0.78 (0.60, 0.96)


*BRIEF scores were reversed so that high values corresponded with high executive functioning. Coeff, log odds coefficient; PP, predicted probability of being in that category or any later category; 1+ RD symptoms, classified as having one, two, three, or four symptoms of RD; 2+ RD symptoms, classified as having two, three, or four symptoms of RD; 3+ RD symptoms, classified as having three or four symptoms of RD; 4 RD symptoms, classified as having all four symptoms of RD; exact *p*-values were reported.*

In the second model, students at the mean, one standard deviation above the mean, and one standard deviation below the mean of Updating Working Memory have a 31%, 26%, and 37% chance, respectively, of being classified as having one, two, three, or four symptoms of RD. Students at the mean, one standard deviation above the mean, and one standard deviation below the mean of Updating Working Memory have a 16%, 13%, and 19% chance, respectively, of being classified as having any two, three, or four symptoms of RD. Students at the mean, one standard deviation above the mean, and one standard deviation below the mean of Updating Working Memory have a 9%, 7%, and 11% chance of being classified as having any three or four symptoms of RD. Finally, all students have a 1% chance of being classified as having four symptoms of RD, regardless of their level of Updating Working Memory ability.

In the third model, students at the mean, at one standard deviation above the mean, and those at one standard deviation below the mean of Shifting have a 31%, 26%, and 38% chance, respectively, of being classified as having one, two, three, or four symptoms of RD. Students at the mean, one standard deviation above the mean, and one standard deviation below the mean of Shifting have a 16%, 12%, and 20% chance, respectively, of being classified as having any two, three, or four symptoms of RD. Students at the mean, at one standard deviation above the mean, and at one standard deviation below the mean of Shifting have a 9%, 7%, and 11% chance, respectively, of being classified as having any three or four symptoms of RD. Finally, all students have a 1% chance of being classified as having four symptoms of RD, regardless of their Shifting performance.

#### Research Question 2: Is One EF Component More Important Than the Others for RD Classification?

Results from the flatness test indicated that the main interaction effect of EF component skill and RD group status was not significant (*F* = 0.68, *p* = 0.6652). This non-significant interaction effect between the three-components of EF and the four RD symptom groups means that the association of EF and RD group status does not depend on which EF component is used as a predictor.

## Discussion

In the present study, we sought to explore the association between EF and RD. Specifically, we examined the link between the three components of EF, consisting of Inhibition, Updating Working Memory, and Shifting (e.g., Miyake et al., 2000), and a hybrid model of RD (Waesche et al., 2011; Spencer et al., 2014; Schatschneider et al., 2016). Although the relation between Updating Working Memory and RD has been extensively explored in the literature (e.g., Sesma et al., 2009), less work has been done examining the relation of Inhibition and Shifting with RD. Additionally, the hybrid model of RD represents the state of the science in RD definition, and there has been no work thus far examining the association of EF and the hybrid model of RD. Our results showed that EF was a significant predictor of RD, and that the probability of RD classification changed based on EF performance. As a second research aim, we pursued the inquiry of whether any one EF more strongly predicted RD within the hybrid model of RD. In doing so, we hoped to determine the EF most likely implicated in deficient reading performance, so it could potentially be targeted in intervention efforts. Our results showed that the number of RD symptoms captured did not vary depending on which EF component was used, and as such, any EF had equal predictive value for RD classification in a hybrid model of RD.

In regards to our first research question, we found that there was a significant relation between all three components of EF (Inhibition, Updating Working Memory, and Shifting) and the hybrid model of RD, no matter how many symptoms of RD the student had. We also found that the chances of being classified as having RD (i.e., having at least one symptom of RD) increased as EF performance worsened, and the chances of RD classification decreased as EF performance improved. Given that reading is a skill that must be taught explicitly in order to be mastered (Gombert, 2003; Vaessen and Blomert, 2010), and that reading skill development has been associated with cognitive control over actions (Gombert, 2003; Shaywitz and Shaywitz, 2008; Bexkens et al., 2015), it is not surprising that EF, our cognitive control system, would play a role in reading acquisition and dysfunction. Previous work has commonly found EF is associated with RD, although the effect size of this association is moderated by RD definition and EF task modality (Stuebing et al., 2002; Booth et al., 2010). Here, we used the hybrid model of RD and a parent-report of EF behaviors and found a significant negative association between EF and RD.

Previous work has suggested that “ANY1PLUS” definition of RD, in which a student has at least one or more symptoms of RD, is the most stable operationalization of RD in predicting future RD symptoms (Spencer et al., 2014). Interestingly, we found that EF’s predictive power of RD was the highest when a student had at least one or more symptoms of RD. This was evidenced by the finding that the predicted probabilities for all three EF components were highest when the outcome being modeled included any combination of hybrid model symptoms (i.e., ANY1PLUS, or any one, any two, any three, or any four symptoms of RD) and were lowest for the outcome that included only the four-symptom RD group. This was likely due to a few possibilities. Either EF is associated with poor reading performance, no matter how it is defined, and/or EF is associated with RD when RD is operationalized in a reliable way, and/or the group with at least one symptom of EF was simply the biggest. This could also be attributable to reasons we cannot establish in the current study.

For our second research question, we conducted a profile analysis in order to explore the differential predictive power of each EF component. We found that Inhibition, Updating Working Memory, and Shifting were all equal predictors of RD. There is considerable conflicting research on the role of each given component of EF with achievement, and less research altogether examining the differential role of each component with RD. Given that no one EF component emerged as a superior predictor, our results point to the idea that EF as a whole (maybe represented as a unitary construct), or any one component of EF, is important in RD identification. We caution that this may be attributable to our use of a single parent-reported measure of EF that used subscales to represent the components. It is likely that the single-reporter measure meant that the correlations between the components of EF were higher than normal, and thus they acted more similarly to each other than task-based measures would demonstrate (e.g., Foy and Mann, 2013). Despite the limitation of the measurement of EF, the BRIEF is a relatively inexpensive, parent-report measure that could be conveniently completed by parents. Moreover, the BRIEF provides a behavioral, instead of a cognitive, index of EF. By virtue of its basis on observable behaviors, the BRIEF is less subject to the task impurity issues that plague most cognitive EF measures (e.g., Miyake et al., 2000) because the behaviors are easier to pinpoint than underlying cognitive processes. In addition, since most investigations of EF and RD use cognitive EF indices (e.g., Altemeier et al., 2008), which correlate poorly with behavioral EF measures (McCauley et al., 2010), our use of a behavioral EF index provides a novel contribution to the research on EF and RD.

Outside of our main research questions directly, we had other interesting findings. Based on our correlations, both Inhibition and Shifting were significantly correlated with all four hybrid model symptoms separately, but Updating Working Memory was only significantly associated with two of the four hybrid model symptoms, namely low word reading achievement and dual-discrepancy RTI in word reading. Therefore, while Inhibition and Shifting would possibly still identify RD in children if single-criterion RD definitions were used, the predictive power of Updating Working Memory in cognitive discrepancy or IQ-achievement discrepancy models (the two symptoms with which it did not significantly correlate) could possibly not fare as well in a single-criterion framework that did not utilize low word reading or RTI definitions. Accordingly, our current study provides evidence for all three EFs as predictors of RD in a hybrid model framework, but does not directly speak to the predictive power of Updating Working Memory when some less comprehensive operationalizations of RD are employed.

An important point to consider is that the current study’s examination of the relation between EF and RD was conducted solely in English, and different relations may have emerged if a more transparent language were used. It is presumed that, as a process, reading acquisition is variable and language-specific (Ziegler and Goswami, 2006). This claim has been corroborated by evidence from neuroimaging studies showing differential brain activation in response to comparable stimuli among different language readers (Ziegler and Goswami, 2006; Holloway et al., 2015). It stands to reason that reading in different languages calls upon different cognitive abilities and their corresponding brain regions, in order to properly respond to cross-linguistic differences in reading demands, like differences in orthographic depth (Gombert, 2003; Ziegler and Goswami, 2006; Holloway et al., 2015). For example, as a language with a deep orthography that is characterized by unpredictable language and speech sound pairs, English may require increased demands on cognitive control to counteract the unpredictable connections between the audio and visual aspects of language when learning to read in English (Holloway et al., 2015). It is not surprising that the present study, which was conducted in English, found a significant relation between EF, the mechanism that enables cognitive control, and reading difficulties. In contrast, learning to read in more shallow orthographies that have transparent language and speech sound pairs, like those found in Dutch and Italian, results in the formation of easier to follow audiovisual rules that make processing more automatic (Holloway et al., 2015). Accordingly, the demands on EF for explicit monitoring and adaptation created by the incongruences in the English language may not exist in a shallow orthography like Dutch, and as a result, students with deficient EF may not display the same RD. To test this possibility, future work should replicate the methods used in the present study using a less orthographically complex language, like Dutch or Italian.

In general, our findings suggest the need for more research to examine the directionality and fundamental nature of the relation between EF and RD as a diagnostic mechanism and, potentially, a way to intervene effectively to reduce the sequelae of RD. We know that EF works as a regulatory system for higher order cognitive processing by enabling the acquisition of new knowledge through the setting, revision, and monitoring of learning-related goals and strategies (Lin et al., 2016), but we are still unsure how this cognitive regulation translates into reading ability and disability. One explanation for the significant association we found between EF and RD is that poor executive functioning overwhelms the cognitive processing system, making reading difficult (Swanson, 2003; Gathercole et al., 2006; Swanson et al., 2006). Without the cognitive resources necessary to choose appropriate strategies to overcome reading difficulties (i.e., setting a time to practice reading daily, choosing an appropriate location to allow concentration when reading, or taking breaks in between reading excerpts in order to mentally review main ideas), children with poor EF may not be able to overcome their reading struggles (Lin et al., 2016). As such, creating learning environments that support EF and self-regulated learning might contribute to stronger reading development (Connor et al., 2010).

Another possibility is that poor reading skills in children with RD result in poor EF through a common third variable that impacts both EF and reading. One such mechanism may be metacognition, whereby children who do not learn to read at an average level also fail to develop effective metacognitive skills, which are vital for the cognitive and self-regulatory processes utilized in EF (Cain et al., 2004; Connor et al., 2016). Recent work also supports the idea of a reciprocal relation between RD and self-regulatory processes, like EF (Connor et al., 2016). For example, children with higher EF abilities may be better able to engage with reading instruction, and together, repeated exposure to such instruction and repeated practice of self-regulation may lead to the enhancement of both reading ability and self-regulatory ability. In the negative direction, it is also possible that poor instruction in reading (i.e., instructionally-induced RD) may also proscribe the development of EF (Vellutino et al., 1996). On the other hand, children with RD may simply also have poor EF skills. Our current study cannot discern this distinction, but future work can begin to differentiate the directionality of these relations.

As an interesting aside, only 2.17% of our sample fell in the four-symptom RD group. Although this group of children was small in absolute numbers, they may, in fact, represent a subset of “inadequate responders” (Toste et al., 2014) or “treatment resisters” (Torgesen, 2000). Torgesen (2000) coined the term “treatment resisters” to describe the 2–6% of children who are resistant to reading intervention, and regardless of targeted preventative efforts, will never reach a “normal” word reading level (Torgesen, 2000). Coincidentally, our sample’s four-symptom RD group falls within this 2–6% resister range, as do the 3% of children that were classified as having only the dual-discrepancy RTI in word reading symptom (see **Table 4**). As such, the treatment resisters may simply be the children that qualify for only the dual-discrepancy RTI in word reading symptom, rather than the children that have all four hybrid model symptoms. This possibility makes sense, as the dual-discrepancy RTI in word reading symptom group captured the children that do not respond to intervention and was also significantly associated with all three EFs. Whether the treatment resisters are the children that have all four RD symptoms or just the dual-discrepancy RTI symptom, these results provide evidence that resisters may not just have reading disabilities, but are likely to have multiple deficits, including poor EF. In fact, students with reading difficulties have been shown to suffer from EF deficits (Fuchs and Fuchs, 2015), and inadequate responders have even been shown to differ from adequate responders in working memory performance (Toste et al., 2014). The hybrid model of RD is not meant to be limited to just the four symptoms used in this study, and adding EF to the model may further reduce measurement error in identifying the students most likely to have RD. In doing so, the extended model that includes EF might support early identification efforts to improve outcomes for children with RD, and possibly, for the most severely reading impaired students as well. This idea was not explicitly tested here, so more future work is needed before conclusions can be drawn.

Although these findings contribute to our understanding of the links between EF and RD, this study is not without limitation. First, our measurement of executive functioning was based on a single parent-report questionnaire, whereas the use of multiple EF measures, including cognitive indices, like the Wisconsin Card Sorting task (Horowitz-Kraus, 2014) or the Stroop task (Miyake et al., 2000), may have increased the reliability of EF scores. Parents may have a skewed concept of their child’s EF abilities, and direct measurement techniques that employ an outside observer could reduce potential biases. On the other hand, the fact that the BRIEF indexes EF based on behavioral manifestations of EF may also be an advantage because it helps avoid the task impurity inherent in verbal EF measures. Second, the questionnaire portion of Project KIDS had an 11.50% recruitment rate from the original intervention projects’ population. Different relationships than those shown in this study may have been revealed by a sample with a greater response rate, as the parents who responded to our questionnaire had children with slightly higher reading and language performance and who were slightly less likely to qualify for free and reduced lunch (indicating higher socioeconomic status). Third, the parent report of EF was measured at a different time point than the language and literacy variables that made up the RD hybrid model. RD classification is relatively stable with the hybrid model (one of the benefits of this model; Schatschneider et al., 2016), but EF undergoes significant developmental changes during these ages (e.g., Anderson, 2002; Bitan et al., 2009), with the component skills of EF continuing to develop along different trajectories until adolescence, when executive control emerges (Anderson, 2002). Therefore, when generalizing our results, we must remain cautious and take into account the fact that parents may have reported on EF abilities that were more developed than they had been at the time of intervention. In order to more closely and accurately test the relation between the EF components and RD classification in a hybrid model, future work should replicate our methods with concurrent EF and achievement data to see if the associations found hold. Another possible consequence of the time elapsed between the original assessment testing and the parent EF ratings is a buildup of frustration due to years of reading difficulties for the students, which they acted out in the form of behaviors measured by the BRIEF (e.g., “talks at the wrong times” or “gets out of control more than friends”; Mahone et al., 2002). Finally, unlike Spencer et al. (2014), no reading fluency measure was included in the calculation of the hybrid model symptoms, and different relations may have been found if a timed reading measure were used.

Not only is RD hard to identify, but it is also one of the most pervasive learning disabilities present in our school systems (Spencer et al., 2014). Children with reading deficiencies encounter myriad of disadvantages, including less practice in developing their reading comprehension skills, a potential for the acquisition of negative views toward reading, and an inability to acquire important knowledge available through print resources (Torgesen, 2000). Although no specific EF component emerged as a superior predictor, this study provides evidence for the overall negative association between all three EF components and RD. As we learn more about the causal mechanisms that underlie RD, including EF, we will be able to contribute to emerging models and theoretical frameworks and design more effective methods for early identification and intervention to help all children, and especially children with RD, succeed in school and throughout their lives.

## Author Contributions

Conceptualization: MD, SH, and CS. Data curation: SH. Formal analysis: MD, SH, and CS. Funding acquisition: SH, CS, CC, and SA. Investigation: MD and SH. Methodology: MD, SH, and CS. Project administration: SH. Resources: SH, CS, CC, and SA. Software: MD and SH. Supervision: SH. Validation: MD and SH. Visualization: MD. Writing original draft: MD, SH, and CS. Writing review and editing: MD, SH, CS, CC, and SA. Final approval: MD, SH, CS, CC, and SA.

## Conflict of Interest Statement

The authors declare that the research was conducted in the absence of any commercial or financial relationships that could be construed as a potential conflict of interest.
